# Physiological effects of adding ECCO_2_R to invasive mechanical ventilation for COPD exacerbations

**DOI:** 10.1186/s13613-020-00743-y

**Published:** 2020-09-29

**Authors:** J.-L. Diehl, L. Piquilloud, D. Vimpere, N. Aissaoui, E. Guerot, J. L. Augy, M. Pierrot, D. Hourton, A. Arnoux, C. Richard, J. Mancebo, A. Mercat

**Affiliations:** 1 Assistance Publique – Hôpitaux de Paris, Hôpital Européen Georges Pompidou, Service de Médecine Intensive - Réanimation, 20 rue Leblanc, 75015 Paris, France; 2 INSERM UMR-S1140, Paris Descartes University, Paris, France; 3 Adult Intensive Care and Burn Unit, University Hospital and University of Lausanne, Lausanne, Switzerland; 4 Medical Intensive Care Unit, University Hospital of Angers, Angers, France; 5 Assistance Publique – Hôpitaux de Paris, Hôpital Européen Georges Pompidou, Unité de Recherche Clinique, Paris, France; 6 Assistance Publique – Hôpitaux de Paris, Service de Médecine Intensive Réanimation, Hôpital de Bicètre, Le Kremlin Bicètre, France; 7 Servei de Medicina Intensiva, Hospital de Sant Pau, Barcelona, Spain

**Keywords:** Extracorporeal carbon dioxide removal, Invasive mechanical ventilation, COPD acute exacerbation, Alveolar ventilation, Work of breathing

## Abstract

**Background:**

Extracorporeal CO_2_ removal (ECCO_2_R) could be a valuable additional modality for invasive mechanical ventilation (IMV) in COPD patients suffering from severe acute exacerbation (AE). We aimed to evaluate in such patients the effects of a low-to-middle extracorporeal blood flow device on both gas exchanges and dynamic hyperinflation, as well as on work of breathing (WOB) during the IMV weaning process.

**Study design and methods:**

Open prospective interventional study in 12 deeply sedated IMV AE-COPD patients studied before and after ECCO_2_R initiation. Gas exchange and dynamic hyperinflation were compared after stabilization without and with ECCO_2_R (Hemolung, Alung, Pittsburgh, USA) combined with a specific adjustment algorithm of the respiratory rate (RR) designed to improve arterial pH. When possible, WOB with and without ECCO_2_R was measured at the end of the weaning process. Due to study size, results are expressed as median (IQR) and a non-parametric approach was adopted.

**Results:**

An improvement in PaCO_2_, from 68 (63; 76) to 49 (46; 55) mmHg, *p* = 0.0005, and in pH, from 7.25 (7.23; 7.29) to 7.35 (7.32; 7.40), *p* = 0.0005, was observed after ECCO_2_R initiation and adjustment of respiratory rate, while intrinsic PEEP and Functional Residual Capacity remained unchanged, from 9.0 (7.0; 10.0) to 8.0 (5.0; 9.0) cmH_2_O and from 3604 (2631; 4850) to 3338 (2633; 4848) mL, *p* = 0.1191 and *p* = 0.3013, respectively. WOB measurements were possible in 5 patients, indicating near-significant higher values after stopping ECCO_2_R: 11.7 (7.5; 15.0) versus 22.6 (13.9; 34.7) Joules/min., *p* = 0.0625 and 1.1 (0.8; 1.4) versus 1.5 (0.9; 2.8) Joules/L, *p* = 0.0625. Three patients died in-ICU. Other patients were successfully hospital-discharged.

**Conclusions:**

Using a formalized protocol of RR adjustment, ECCO_2_R permitted to effectively improve pH and diminish PaCO_2_ at the early phase of IMV in 12 AE-COPD patients, but not to diminish dynamic hyperinflation in the whole group. A trend toward a decrease in WOB was also observed during the weaning process.

*Trial registration *ClinicalTrials.gov: Identifier: NCT02586948.

## Background

Chronic obstructive pulmonary disease (COPD) is currently the fourth leading cause of death in the U.S. and is expected to become the third leading cause of death [1]. Value of non-invasive ventilation (NIV) for severe AE- COPD was formally demonstrated by randomized clinical trials [2, 3]. While the hospital mortality of patients successfully treated with NIV has decreased over years, and is currently less than 10%, mortality in patients requiring IMV after NIV failure is close to 30% [4]. Among the techniques which could help to improve the prognosis of such patients, extracorporeal CO_2_ removal (ECCO_2_R) seems to be a very promising approach [5, 6]. However, most of the studies focused on ECCO_2_R in NIV AE-COPD patients, with the aim to prevent intubation [7–9] or to provide an additional respiratory support after extubation [10]. Only a small number of IMV COPD patients were studied under ECCO_2_R, with the aim to facilitate extubation [10–13]. ECCO_2_R was initiated early after intubation in 2 studies [12, 13], while the delay between intubation and ECCO_2_R initiation was higher than 15 days in another study [11]. We preliminarily reported an ECCO_2_R-induced reduction in work of breathing and CO_2_ production in such a setting [14], confirming and extending previous observations [15].

In the present study, we hypothesized that the addition of ECCO_2_R at the early phase of IMV could both improve gas exchanges and could also permit to diminish respiratory rate (RR), therefore, minimizing dynamic hyperinflation in AE-COPD patients. Beyond efficacy assessments, we also planned to describe the complications or adverse events associated with the technique, since bleeding and clotting complications were frequently reported in AE-COPD patients [7].

## Materials and methods

### Study design

This interventional open prospective study was planned to recruit 12 deeply sedated IMV AE-COPD patients in tertiary-level ICUs in France. An institutional ethic board (Comité de Protection des Personnes Ile-de-France VI, Paris, France) approved the protocol (protocole EPHEBE P141 203-ID CRB: 2015-A100446-43). Informed consent was obtained from patients' legal representatives. The study was prospectively registered in ClinicalTrials.gov: Identifier: NCT02586948.

### Patients

Consecutive COPD patients older than 18 yrs. hospitalized for hypercapnic respiratory failure requiring IMV were prospectively screened for inclusion in the study. Inclusion criteria were:

- AE of a known or suspected COPD
- Intubation (whatever the reason for intubation which had to be specified)
- MV since less than 72 h.
- Persistent respiratory acidosis and hyperinflation, while the patients were deeply sedated and paralysed
- Written inform consent obtained from patient’s legal surrogate

Criteria for persistent respiratory acidosis and hyperinflation were the combination of: pH < 7.30, PaCO_2_ > 55 mm Hg and intrinsic PEEP (PEEPi) (end-expiratory occlusion) > 5 cmH_2_O, while on assist-controlled volume ventilation with the following settings: V_T_: 8 mL/kg of predicted body weight (PBW), RR: 12/min., applied PEEP: 0 cmH_2_O, I/E ratio: 1/3. Non-inclusion criteria were as follows: Body Mass Index (BMI) > 35 kg/m^2^, PaO_2_/FiO_2_ < 200 mm Hg, history of haemorrhagic stroke, history of heparin-induced thrombocytopenia and any current severe bleeding. The protocol of the study was explained to the legal representatives and informed consent was obtained from patients legal representatives. When possible, the same explanations were further provided to the patient himself after full recovery, for obtaining a definitive post hoc written consent.

### Medical devices

The Hemolung® ECCO_2_R system (Alung Technologies, Pittsburgh, PA) was used. It consists of an exchange cartridge (membrane surface 0.59 m^2^) which, in connection with a controller and tubing, ensures ECCO_2_R of about 80 mL/min. at extracorporeal blood flow rates comprised between 350 and 550 mL/min. The vascular access is achieved by means of a double lumen 15.5 F central venous catheter. The maximum duration of use of the circuit, as specified by the manufacturer, is 7 days. Anticoagulation was achieved by the mean of continuous unfractionated heparin infusion aiming to obtain daily therapeutic antiXa activities between 0.3 and 0.6 UI/mL. No systematic daily measurement of plasma free hemoglobin was performed during the study.

The CareScape R860 ventilator (General Electric Healthcare) was used allowing continuous measurement of the native lung's VCO_2_ and serial measurements of the functional residual capacity (FRC) (applied PEEP set at zero) or end-expiratory lung volume (EELV) (any positive applied PEEP) using the nitrogen washout/washin technique [16, 17]. A Nutrivent catheter (Sidam, Mirandola, Italy) was inserted for esophageal pressure measurements, allowing the calculation of inspiratory work of breathing (WOB) during the weaning process as previously described [14].

### Protocol of the study

**Fig. 1:**
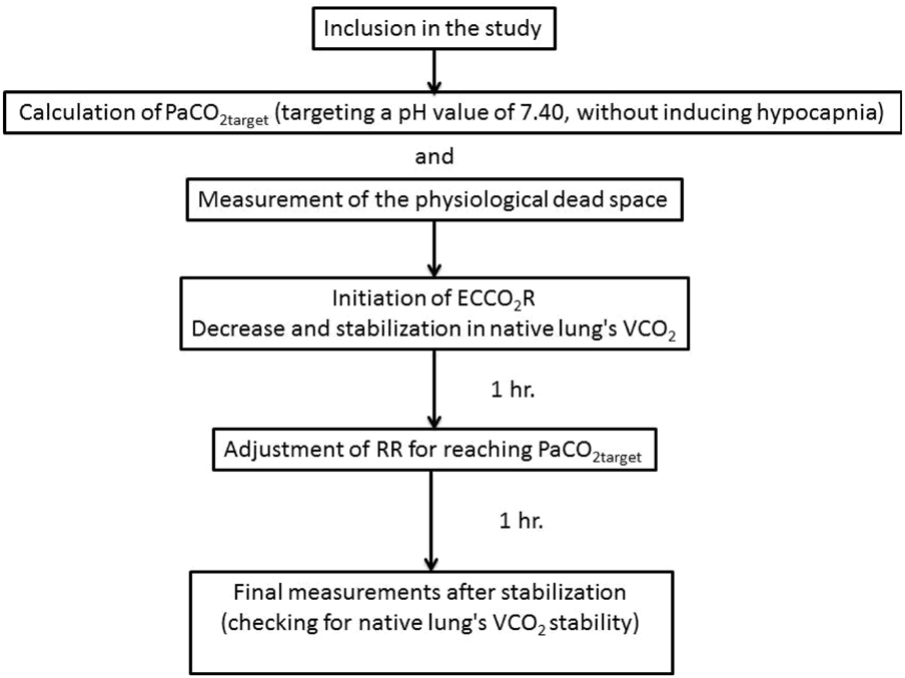
Flowchart of the study. PaCO_2target_: PaCO_2_ corresponding to a pH value of 7.40, based on the Henderson-Hasselbach equation, governing the relationship between the PaCO_2_, pH and bicarbonates plasma values. In cases of mixed respiratory and metabolic acidosis, a PaCO_2target_ value of 40 mmHg was retained. RR: respiratory rate

Figure 1 illustrates the flowchart of the study.

After inclusion in the study, we first calculate the target PaCO_2_ (PaCO_2target_) corresponding to a pH value of 7.40, based on the Henderson-Hasselbach equation governing the relationship between PaCO_2_, pH and bicarbonates plasma values. In cases of mixed respiratory and metabolic acidosis, any PaCO_2target_ below the normal PaCO_2_ value was replaced by the 40 mmHg value.

The second step of the study was to measure the physiological dead space (*V*_D_) using the Bohr-Enghoff equation: *V*_D_/*V*_T_ = (PaCO_2_ – P_E_CO_2_)/PaCO_2_.

The third step of the study was to start ECCO_2_R. After cannulation and initiation of the treatment, an increase in the sweep gas flow (using pure O_2_) generally up to 10 L/min. induced a decrease in native lung's VCO_2_. We checked for stabilization of the latter, with a delay of 1 h.

The fourth part of the study was then to adjust RR for reaching PaCO_2target_. For that purpose, we used the proportionality equation between alveolar ventilation, native lung's VCO_2_ and PaCO_2_: (*V*_T_ – *V*_D_) × RR = (K × VCO_2_)/PaCO_2_; 

expressed as:

$$ {\text{RR }} = \, \left( {K \, \times {\text{ VCO}}_{{2}} } \right) \, /[{\text{PaCO}}_{{{\text{2target}}}} \times \, \left( {V_{{\text{T}}} {-} \, V_{{\text{D}}} } \right)] $$

assuming that *V*_D_ was unchanged during the study.

The fifth part of the study was to perform final measurements after waiting again for stability of the native lung's VCO_2_, with a further delay of 1 h. If required, we adjusted the extracorporeal blood flow and/or sweep gas flow with the aim to keep unchanged the native lung's VCO_2_ after the initial decrease.

### Study endpoints

The primary outcome measure was PEEPi, measured during a prolonged expiratory pause at inclusion in the study and after initiation of ECCO_2_R combined with RR adjustment. We choose PEEPi as the primary outcome measure because we assumed that improvement in arterial pH and PaCO_2_ would be obvious and that the medical device would be powerful enough for achieving both improvements in respiratory acidosis and in dynamic hyperinflation. Secondary end-points measured within the same time frame were: plateau pressure, peak pressure (Ppeak), FRC, PaCO_2_, PaO_2_, arterial pH, hemoglobin saturation (SatHbO_2_), extracorporeal VCO_2_, standard hemodynamic parameters. We also calculated *V*_T_/*T*_E_ as a major determinant of dynamic hyperinflation.

Based on recorded files, WOB at the end of the weaning process was measured just before extubation with and without ECCO_2_R under low Pressure Support Ventilation as previously described [14]. As a supplemental analysis, we also pooled the WOB results of the present study with previously published results of 2 pilot patients obtained using the same experimental design [14]. ECCO_2_R-related adverse events were recorded during the whole ICU-stay. This included severe hemolysis defined as a serum free hemoglobin level higher than 500 mg/L and/or association to jaundice, hemoglobinuria or impaired renal function. Time on ECCO_2_R, time on IMV, length of stay in ICU and in hospital and mortality at 28 days were recorded.

### Sample size calculation and statistical analysis

Considering results obtained in preliminary pilot patients, we hypothesized a mean value of PEEPi at inclusion of 9 cmH_2_O along with an average reduction of 2 cmH_2_O of PEEPi after initiation of ECCO_2_R combined with RR adjustment (SD pooled = 1.9- slightly below the average reduction). Based on these assumptions, with 12 evaluable patients, a paired *t*-test would reach a statistical power of 90% to conclude to the statistical significance of the difference before/after ECCO_2_R at the (two-sided) alpha level = 0.05 (nQuery MOT1 module).

Demographics and clinical characteristics of included patients at inclusion were described as follows: quantitative and qualitative variables were tabulated with medians, interquartile range (IQR) and range (min; max), and counts and proportions, respectively. We secondly described primary and secondary endpoints, at each time point, with the same statistical indicators. Results are expressed in the results sections as median (IQR). Due to study size, a non-parametric approach was adopted. For principal analysis on primary endpoint, we implemented Wilcoxon signed-rank test to compare PEEPi at inclusion and PEEPi after initiation of ECCO_2_R combined with RR adjustment. Regarding secondary endpoints, we performed the same test as for primary endpoint. For endpoints assessed several times, graphs representing variable distributions at each timepoint helped interpreting statistical parameters and tests. In this exploratory trial, statistical significance for p-values was fixed to 0.05 for all statistical tests. We summarized SAEs by number (frequency) of patients to whom SAE occurred. The software used for analyses of data was SAS (r) Proprietary Software 9.4. (SAS Institute Inc., Cary, NC).

## Results

Twelve patients were recruited during an 18-month period in 2 centers. Table 1 shows characteristics at inclusion. Causes of AE were viral pulmonary infections in 5 patients, bacterial pulmonary infection in 4 patients, pneumothoraxes in 2 patients (all with successful pleural drainage at the time of measurement), and exacerbation in a post-surgical context for the last patient.

**Table 1:** Characteristics of the 12 patients at inclusion

Clinical variable	Result
Age (years)	65 (56.5; 73.5)
Female/male: *n*/*n*	4/8
SAPS II	33 (28.5; 39.5)
Body mass index (kg/m^2^)	25.2 (23.7; 28.3)
NIV failure as the reason for intubation: *n* (%)	12 (100%)
Home NIV before admission: *n* (%)	3 (25%)
Long-term oxygen therapy before admission: *n* (%)	2 (17%)

Results are expressed as median (IQR) or number of patients (%)

**Fig. 2:**
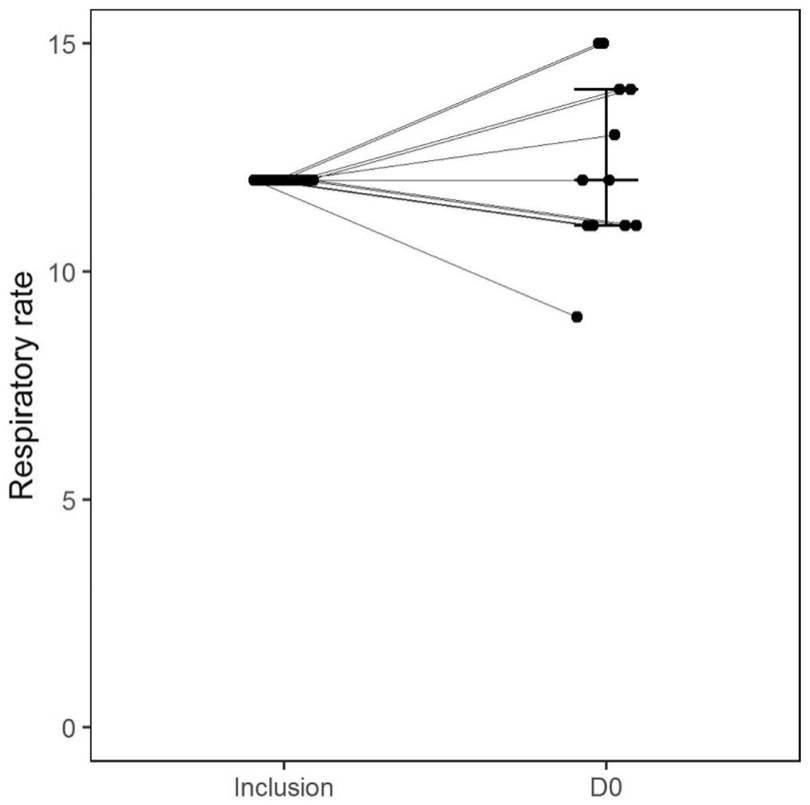
Respiratory rate before ECCO_2_R initiation and after ECCO_2_R initiation and adjustment aiming to improve arterial pH value. D0: first day with ECCO_2_R, after adjustment of respiratory rate aiming to improve arterial pH value

After initiation of ECCO_2_R, the RR adjustment algorithm (aiming to improve arterial pH value) resulted in RR decrease in 5 patients, in RR increase in 5 patients, while RR was maintained unchanged in the remaining 2 patients (Fig. 2). As a consequence, median minute ventilation was not modified, from 6300 (5112; 6900) to 6300 (4800; 6725) mL/min., *p* = 0.8457. PEEPi after initiation of ECCO_2_R and RR adjustment was not significatively different from basal values: 8.5 (7.0; 10.0) to 8.0 (5.5; 9.5) cmH_2_O, *p* = 0.1191. Other respiratory parameters (mechanical ventilator settings, other parameters of hyperinflation, ABG values and native lungs VCO_2_ values) before ECCO_2_R initiation and after ECCO_2_R initiation combined with RR adjustment are mentioned in Table 2, in Additional file 1: Fig. S1 (gas exchanges parameters) and Additional file 1: Fig. S2 (ventilatory parameters). In the 7 patients with pure respiratory acidosis before ECCO_2_R initiation, we found that the RR adjustment in addition to ECCO_2_R led to increase in arterial pH from 7.27 (7.25; 7.30) to 7.40 (7.35; 7.43). Median extracorporeal blood flow was 460 (430; 505) mL/min., with a median sweep gas flow of 10 (10; 10) L/min. Median extracorporeal VCO_2_ was 85 (80–89) mL/min. No variations in hemodynamic parameters were observed without or with ECCO_2_R.

**Table 2:** Respiratory parameters before ECCO_2_R and after ECCO_2_R initiation combined with RR adjustment

	Without ECCO_2_R	ECCO_2_R with RR adjustment	*p*
RR (/min.)	12 (12; 12)	12 (11; 14)	0.4236
*V*_T_ (mL/Kg PBW)	8.0 (8.0; 8.0)	8.0 (8.0; 8.0)	1
FiO_2_	32.5 (30; 40)	35 (30; 40)	0.75
PEEPi (cmH_2_0)	8.5 (7.0; 10.0)	8.0 (5.5; 9.5)	0.1191
Pplateau (cmH_2_0)	15.5 (14.0; 17.5)	16.0 (14.0; 17.5)	0.6323
Ppeak (cmH_2_O)	42.0 (37.5; 49.5)	41.5 (37.5; 51.5)	0.6323
FRC (mL)	3544 (1908; 4849)	2830 (2066; 3818)	0.3013
*V*_T_/*T*_E_ (mL/sec.)	140 (114; 153)	140 (107; 149)	0.8457
PaCO_2_ (mmHg)	68 (63; 76)	49 (46; 55)	0.0005
PaO_2_ (mmHg)	73 (60; 85)	78 (69; 94)	0.1831
pH	7.25 (7.23; 7.29)	7.35 (7.32; 7.40)	0.0005
SatHbO_2_ (%)	93 (88; 95)	96 (95; 97)	0.0337
VCO_2_resp (mL/min.)	203 (150; 243)	121 (101; 155)	0.0015

*RR* respiratory rate, *V*_*T*_ tidal volume, *PBW* predicted body weight, *PEEPi* intrinsic Positive End Expiratory Pressure, *Pplateau* plateau pressure, *Ppeak* peak pressure, *FRC* functional residual capacity, *V*_*T*_*/T*_*E*_ ratio of tidal volume by expiratory time; *PaCO*_*2*_ arterial partial pressure in carbon dioxide, *PaO*_*2*_ arterial partial pressure in oxygen, *SatHbO*_*2*_ Oxygen hemoglobin saturation, *VCO*_*2*_*resp* native lungs' CO_2_ elimination

Results are expressed as median (IQR)

Median ECCO_2_R duration was 5.55 (3.10; 7.25) days. Median sweep gas flow was 10 L/min. from day 1 to day 6. Additional file 1: Fig. S3 illustrates the course of total PEEP and EELV under ECCO_2_R until day 4. Of note, an external positive PEEP (generally between 5 and 8 cmH_2_O) was set after stopping deep sedation beyond the first days of IMV, to favor the synchronization between the patient and the mechanical ventilator and to counteract flow limitation. Additional file 1: Fig. S4 illustrates the course of ABG parameters and Additional file 1: Fig. S5 illustrates the course of hematological parameters under ECCO_2_R until day 7. Mainly, a mild thrombocytopenia was observed in the whole group.

Inspiratory WOB measurements with and without ECCO_2_R were possible in only 5 patients during the weaning process, due to premature cessation of ECCO_2_R before readiness of patients to perform a low Pressure Support Ventilation trial in 6 patients (mainly in relation with hemorrhagic and thrombotic complications) and due to accidental removal of the Nutrivent probe in one patient. WOB measurements were performed in conscious patients while breathing at a low pressure support level with ECCO_2_R and after switching the sweep gas flow from current value to 0 L/min. for a 1 h period. Results are indicated in Table 3. Results adding the previously published results of 2 pilot patients using a similar design are presented as Additional file 1: Table S1.

**Table 3:** Work of breathing (WOB) measurements in 5 patients with and without ECCO_2_R

	ECCO_2_R+	ECCO_2_R−	*p*
WOB (J/L)	1.10 (0.8; 1.40)	1.50 (0.9; 2.80)	0.0625
WOB (J/min.)	11.70 (7.50; 15.00)	22.60 (13.90; 34.70)	0.0625
WOB (J/breath)	0.59 (0.39; 0.79)	0.94 (0.56; 1.29)	0.0625
RR (/min.)	19 (19; 20)	24 (24; 25)	0.1250
VCO_2_tot (mL/min.)	308 (307; 347)	321 (312; 417)	0.3125
VCO_2_resp (mL/min.)	242 (240; 280)	321 (312; 417)	0.0625

*ECCO*_*2*_*R+* treatment with ECCO_2_R while breathing at a low pressure support level, *ECCO*_*2*_*R−* after switching the sweep gas flow to 0 L/min. for 1 h, WOB: work of breathing expressed as Joules per liter of minute ventilation (J/L) or as Joules per breath (J/breath), *VCO*_*2*_*tot* whole body CO_2_ elimination, *VCO*_*2*_*resp* native lungs CO_2_ elimination

Results are expressed as median (IQR)

Three patients died in-ICU and 9 were successfully discharged from ICU and hospital. The causes of death were one hemorrhagic stroke during ECCO_2_R therapy and 2 septic shocks in relation with ventilator-associated pneumonia. The median IMV total duration was 8 (6; 18) days. The median IMV duration after ECCO_2_R initiation was 6 (4; 16.5) days. The median ICU-stay duration was of 14.5 (8–22.5) days. The median hospital length of stay was 39 (18.5; 73) days. A ventilator-associated pneumonia was diagnosed in 4 patients. Three hemorrhagic complications were observed during ECCO_2_R therapy, including one fatal hemorrhagic stroke (in the absence of any unfractionated heparin overdosing or thrombocytopenia). Three thrombotic complications were observed (2 ECCO_2_R catheter thrombosis, one ECCO_2_R circuit thrombosis). No patient suffered from severe clinical hemolysis. We didn't observe air bubble in the circuit in any patient.

## Discussion

We report a physiological and clinical evaluation of a low-to-middle extracorporeal blood flow veno-venous ECCO_2_R system in 12 very severe AE-COPD patients studied shortly after intubation. Severity of the patients was assessed by the combination of respiratory acidosis and elevated intrinsic PEEP under pre-specified respiratory settings aimed to avoid excessive dynamic hyperinflation in deeply sedated IMV patients. Moreover, all patients were intubated after NIV failure. Dynamic hyperinflation was also assessed by FRC and EELV measurements using the nitrogen washin-washout method, providing original results in this specific COPD population. Indeed, such patients were not included or were excluded from previous studies [18]. As expected, we observed very high baseline FRC values as compared to published reference values measured in the supine position [19].

Initiation of ECCO_2_R was associated with a median extracorporeal CO_2_ removal amount of 85 mL/min., corresponding to 42% of the pre-ECCO_2_R whole body CO_2_ production. Accordingly, there was a decrease in native lungs' CO_2_ elimination, which, in conjunction with RR adjustment, permitted to improve arterial pH and to obtain a median absolute decrease in PaCO_2_ of 19 mmHg. This could be beneficial at the early stage of IMV in AE COPD patients, mainly by minimizing the deleterious effects of acute hypercapnia on ventilator demands, therefore, allowing to shorten deep sedation periods and to rapidly initiate the IMV weaning process. We didn't observe any ECCO_2_R-induced deleterious effect on oxygenation, as sometimes mentioned in COPD patients [9, 10, 20]. However, severely hypoxemic patients were excluded from our study. Moreover, we used a low-to-middle blood flow ECCO_2_R device, therefore, minimizing the ECCO_2_R-induced imbalance between native lung's VO_2_ and VCO_2_ [20]. We also found a higher SatHbO2 under ECCO_2_R, which could at least in part be explained by a left shift of the O_2_ dissociation curve due to a decrease in arterial PaCO_2_ and to a parallel increase in arterial pH. Although probably too complex for a general clinical use, the algorithm for RR adjustment performed well for arterial pH improvement. Such a result was favored by the hemodynamic stability of the patients during ECCO_2_R initiation associated with stability in whole body CO_2_ production. By choice, we didn't retain an algorithm based on *V*_T_ reduction. This was based on the fact that the absolute value of physiological dead space for CO_2_ depends of the absolute value of *V*_T_, therefore, allowing easier calculations when keeping a fixed absolute *V*_T_ value [21].

However, despite the use of quasi-maximal extracorporeal blood and sweep gas flows, the algorithm led to a decrease in RR in only 5 patients. This explains that no improvement in PEEPi, as the primary outcome measure, was observed in the whole group. The clinical correlate is that the ECCO_2_R system was not able in our group of very severe IMV COPD patients to *both* improve respiratory acidosis and improve dynamic hyperinflation. However, it’s obvious that alternative adjustments algorithms would have been associated with different results. As an example, it could have been possible to first reduce RR and V_T_ after ECCO_2_R initiation while keeping PaCO_2_ at the same level. Such a strategy very probably would have been associated with a significant decrease in PEEPi. Moreover, in the clinical setting, clinicians will have the possibility to tailor personalized strategies: by simply choosing different PaCO_2_ target and by calculating individual RR adjustment, clinicians have the possibility to arbitrate between respiratory acidosis and dynamic hyperinflation respective improvements. It's also likely that ECCO_2_R systems allowing higher extracorporeal CO_2_ removal amounts could have been associated with higher improvements in hyperinflation parameters and in respiratory acidosis. Altogether, this illustrates the need for clinicians to develop clinical strategies of ECCO_2_R initiation in deeply sedated IMV COPD patients. Such strategies should be based on the severity of patients, mainly assessed by parameters of dynamic hyperinflation and respiratory acidosis. Based on animal and clinical studies, clinicians should also take into account the performances of the different ECCO_2_R devices and their effects on native lungs respiratory CO_2_ elimination [22, 23]. Providing such strategies could have important implications for the care of patients and for the design of future RCTs aiming to prove important clinical benefits of ECCO_2_R in very severe AE-COPD patients. In addition, we have to mention that our algorithm is not per se suitable for awake patients. This point is important, since ECCO_2_R can be proposed in AE-COPD patients at high risk of NIV failure, or in cases of difficult IMV weaning. Finally, such low-to-intermediate extracorporeal blood flow devices could be viewed as more suitable for paralyzed moderate ARDS patients with minimal CO_2_ production rather than for very severe AE-COPD patients.

In line with PEEPi results, FRC and *V*_T_/*T*_E_ were not significantly improved in the whole group. One could question the validity of FRC measurements in patients treated by ECCO_2_R, since ECCO_2_R can modify the native lung’s respiratory quotient [20]. However, the nitrogen fraction calculation is based on direct measurements of both O_2_ and CO_2_ fractions when F_i_O_2_ is lower than 65%, as indicated by the manufacturer [16]. Since our study included only non-severely hypoxemic patients, with FiO_2_ < 65%, we are confident in the validity of our results. Also, the course of FRC results was coherent with PEEPi results.

We previously reported an ECCO_2_R-induced benefit in terms of breathing pattern and of work of breathing in 2 IMV AE-COPD at the end of the weaning process [14]. Using the same design, we observed similar trends in 5 patients. Considering a possible lack of statistical power due to the number of patients, we pooled the results of the 2 studies and observed significantly less WOB (expressed either in Joules per min, per liter of ventilation or per breath) under ECCO_2_R. However, since we cannot exclude selection bias, these results are presented with great caution and should not be extrapolated to clinical practice. Such results obtained in non-sedated patients only suggest that ECCO_2_R could favor a more rapid liberation of IMV, as compared to standard care of IMV AE-COPD patients [5, 6, 15]. Moreover, the fact that efficiency of ECCO_2_R was observed several days after initiation, could open the way for further studies of different clinical strategies for ECCO_2_R weaning.

The median duration of ECCO_2_R was near to the maximal duration of the circuit as indicated by the manufacturer. Such result is important to consider for the choice of ECCO_2_R devices and circuits in COPD patients. We observed one fatal intracerebral bleeding. Such fatality, along with other hemorrhagic complications and thrombosis, illustrate the need to improve the knowledge of the interaction between ECCO_2_R circuits, anticoagulation regimen and coagulation system of the patients. Indeed, hemorrhagic complications can be favored by an usual mild thrombocytopenia as observed in our study and by other factors such as the occurrence of an acquired Willebrand disease, as previously preliminary reported with the Hemolung system [24] and such as a severe endothelial dysfunction, as recently reported by our group [25]. Moreover, fewer side effects could also be expected with higher extracorporeal blood flow devices, as recently shown in ARDS patients [26]. Nevertheless, the in-hospital mortality rate was found to be lower than the mortality rate observed in IMV AE-COPD patients by Burki et al. with the same device, which could suggest a benefit to initiate ECCO_2_R early in the course of IMV in COPD patients [11].

One of the main limitations of the study was a too optimistic hypothesis at the time of conception of the study, leading to an overestimation of the ability of Hemolung device for CO_2_ removal in such severe AE-COPD patient [11, 14]. Another limitation was the choice to use standardized mechanical ventilator settings, as part of our usual respiratory bundle in such severe AE-COPD patients. It is, therefore, conceivable that more personalized settings could have been more appropriate for certain patients. One other limitation was the assumption of an unchanged *V*_D_/*V*_T_ during all points of the study. Indeed, there was a possibility of individual decrease (or increase) in *V*_D_/*V*_T_ in patients with decrease (or increase) in RR. Such variations in *V*_D_/*V*_T_ after limited modifications in ventilatory settings have been reported previously in AE-COPD patients [27]. However, there were no differences in the whole group between PEEPi, plateau pressure, Ppeak and EELV values at baseline and after initiation of ECCO_2_R combined with RR adjustments. The lack of standard of care control group was also a limit of the study for evaluating dynamic hyperinflation independently of ventilation on a more prolonged time. Accordingly, the different initial time points were separated by a delay of 1 h. Therefore, we cannot exclude that a more delayed ECCO_2_R-induced improvement in regional ventilation could have occurred and allowed decreasing RR, I/E ratio or *V*_T,_ all important determinants of dynamic hyperinflation. We didn't observed severe hemolysis in contrast to other reports [26, 28]. However, the observation is limited by the lack of systematic daily plasma free hemoglobin measurement, which is now a standard practice in our centers. The low inclusion rate of the study and the fact that WOB measurements were not possible for the majority of included patients are also clear limitations.

## Conclusions

Using a formalized protocol of RR adjustment, ECCO_2_R permitted to effectively improve pH and diminish PaCO_2_ at the early phase of IMV in 12 AE-COPD patients, but not to diminish dynamic hyperinflation in the whole group. Such results could support the clinical implementation of fine-tuned algorithms derived from our protocol taken into account the 2 main goals of ECCO_2_R at the early phase of IMV, i.e., controlling both hyperinflation and respiratory acidosis.

## Supplementary information

**Additional file 1: Table S1.** Work of breathing (WOB) measurements in 7 patients with and without ECCO_2_R. **Figure S1.** Gas exchanges parameters before ECCO_2_R initiation and after ECCO_2_R initiation and adjustment aiming to improve arterial pH value. **Figure S2.** Ventilatory parameters before ECCO_2_R initiation and after ECCO_2_R initiation and adjustment aiming to improve arterial pH value. **Figure S3.** Daily course of total PEEP and EELV under ECCO_2_R until day 4. **Figure S4.** Daily course of ABG parameters under ECCO_2_R until day 7. **Figure S5.** Course of hematological parameters under ECCO_2_R until day 7.

## Data Availability

The datasets used and/or analysed during the current study are available from the corresponding author on reasonable request.
