# Supplementary material for: Physiological effects of adding ECCO2R to invasive mechanical ventilation for COPD exacerbations
Source: Ann Intensive Care. 2020 Sep 29;10:126. doi: 10.1186/s13613-020-00743-y (PMC7523267; doi:10.1186/s13613-020-00743-y)
Supplement: Supplementary file 1 — Additional file 1: Table S1. Work of breathing (WOB) measurements in 7 patients with and without ECCO2R. Figure S1. Gas exchanges parameters before ECCO2R initiation and after ECCO2R initiation and adjustment aiming to improve arterial pH value. Figure S2. Ventilatory parameters before ECCO2R initiation and after ECCO2R initiation and adjustment aiming to improve arterial pH value. Figure S3. Daily course of total PEEP and EELV under ECCO2R until day 4. Figure S4. Daily course of ABG parameters under ECCO2R until day 7. Figure S5. Course of hematological parameters under ECCO2R until day 7. [file 13613_2020_743_MOESM1_ESM.docx]

# Physiological effects of adding ECCO_2_R to invasive mechanical ventilation for COPD exacerbations

**Electronic supplementary material**

- Table 1esm: Work of breathing (WOB) measurements in 7 patients with and without ECCO_2_R.
- Figure 1esm: Gas exchanges parameters before ECCO_2_R initiation and after ECCO_2_R initiation and adjustment aiming to improve arterial pH value.
- Figure 2esm: Ventilatory parameters before ECCO_2_R initiation and after ECCO_2_R initiation and adjustment aiming to improve arterial pH value.
- Figure 3esm: Daily course of total PEEP and EELV under ECCO_2_R until Day 4.
- Figure 4esm: Daily course of ABG parameters under ECCO_2_R until Day 7.
- Figure 5esm: Course of hematological parameters under ECCO_2_R until Day 7.

Table 1esm: Work of breathing (WOB) measurements in 7 patients with and without ECCO_2_R.

|  | ECCO_2_R + | ECCO_2_R - | p |
| --- | --- | --- | --- |
| WOB (J/L) | 1.10 (0.5;1.5) | 1.50 (0.7;2.8) | 0.0156 |
| WOB (J/min.) | 11.70 (2.4;15.0) | 17.5 (7.7;34.7) | 0.0156 |
| WOB (J/breath) | 0.59 (0.14;0.79) | 0.80 (0.35;1.29) | 0.0156 |
| RR (/min.) | 19 (17;21) | 24 (22;27) | 0.0625 |
| VCO_2_tot (mL/min.) | 307 (270;347) | 312 (310;417) | 0.0781 |
| VCO_2_resp (mL/min.) | 240 (190;280) | 312 (310;417) | 0.0156 |

Abbreviations: ECCO_2_R +: treatment with ECCO_2_R while breathing at a low pressure support level, ECCO_2_R -: after switching the sweep gas flow to 0 L/min. for one hour, WOB: work of breathing expressed as Joules per liter of minute ventilation (J/L) or as Joules per breath (J/breath), VCO_2_tot: whole body CO_2_ elimination, VCO_2_resp: native lungs CO_2_ elimination.

Results are expressed as median (IQR).

Figure 1esm: Gas exchanges parameters before ECCO_2_R initiation and after ECCO_2_R initiation and adjustment aiming to improve arterial pH value.


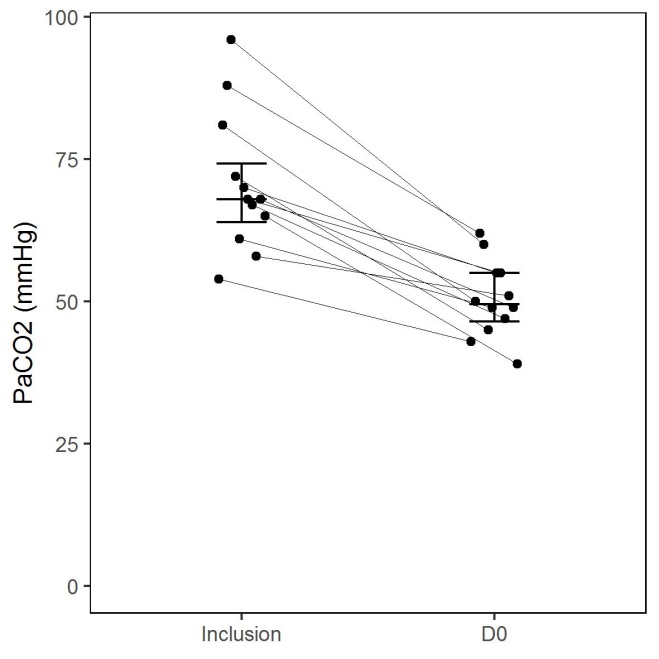

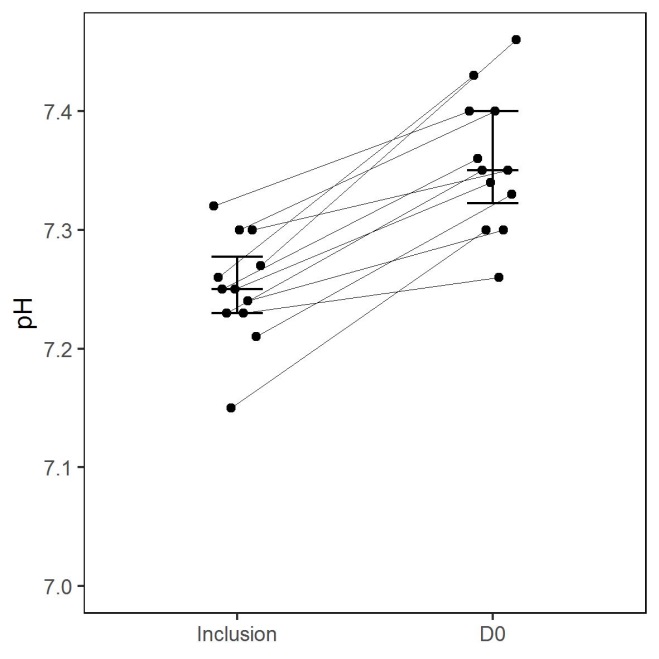


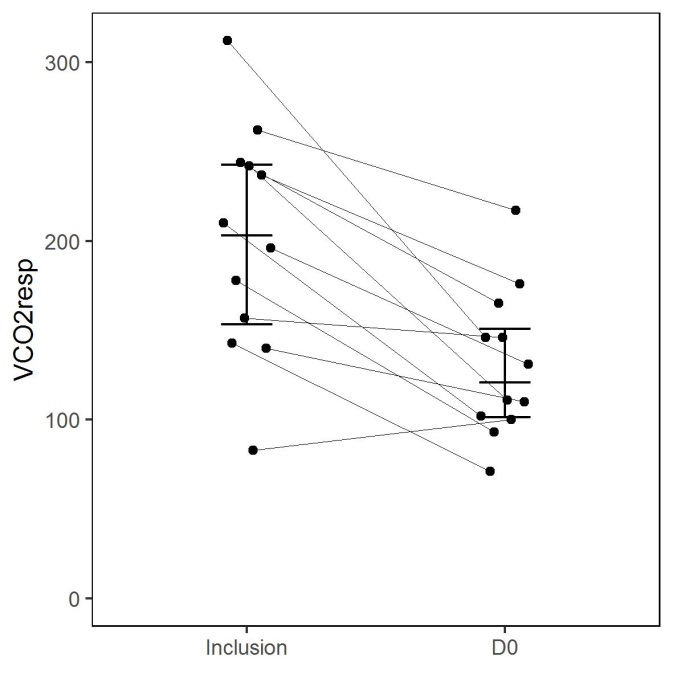


Figure 2esm: Ventilatory parameters before ECCO_2_R initiation and after ECCO_2_R initiation and adjustment aiming to improve arterial pH value.


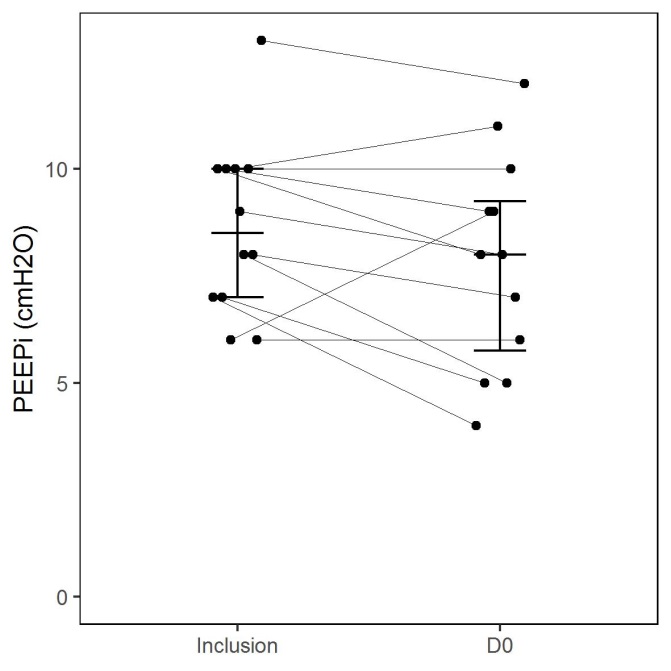

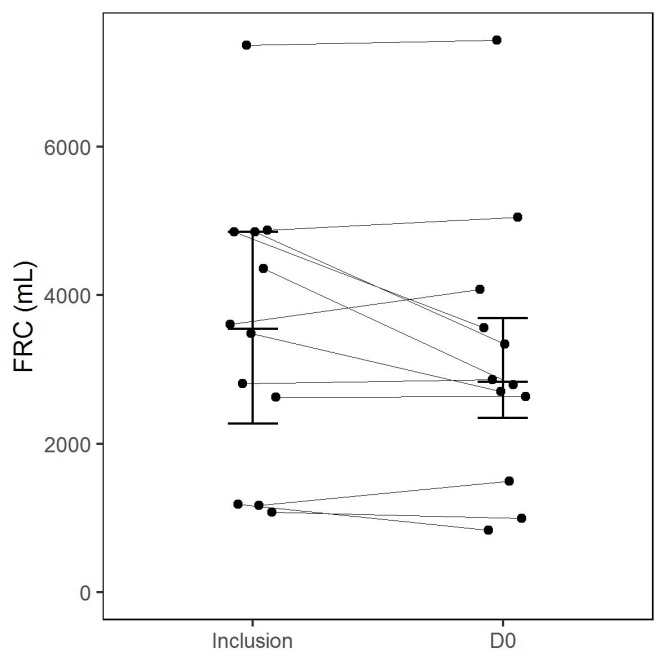


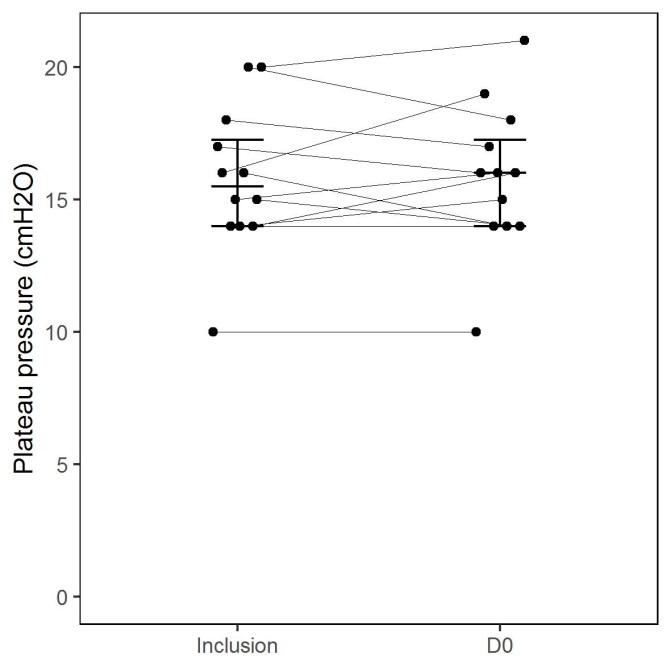


Figure 3esm: Daily course of total PEEP and EELV under ECCO_2_R until Day 4.


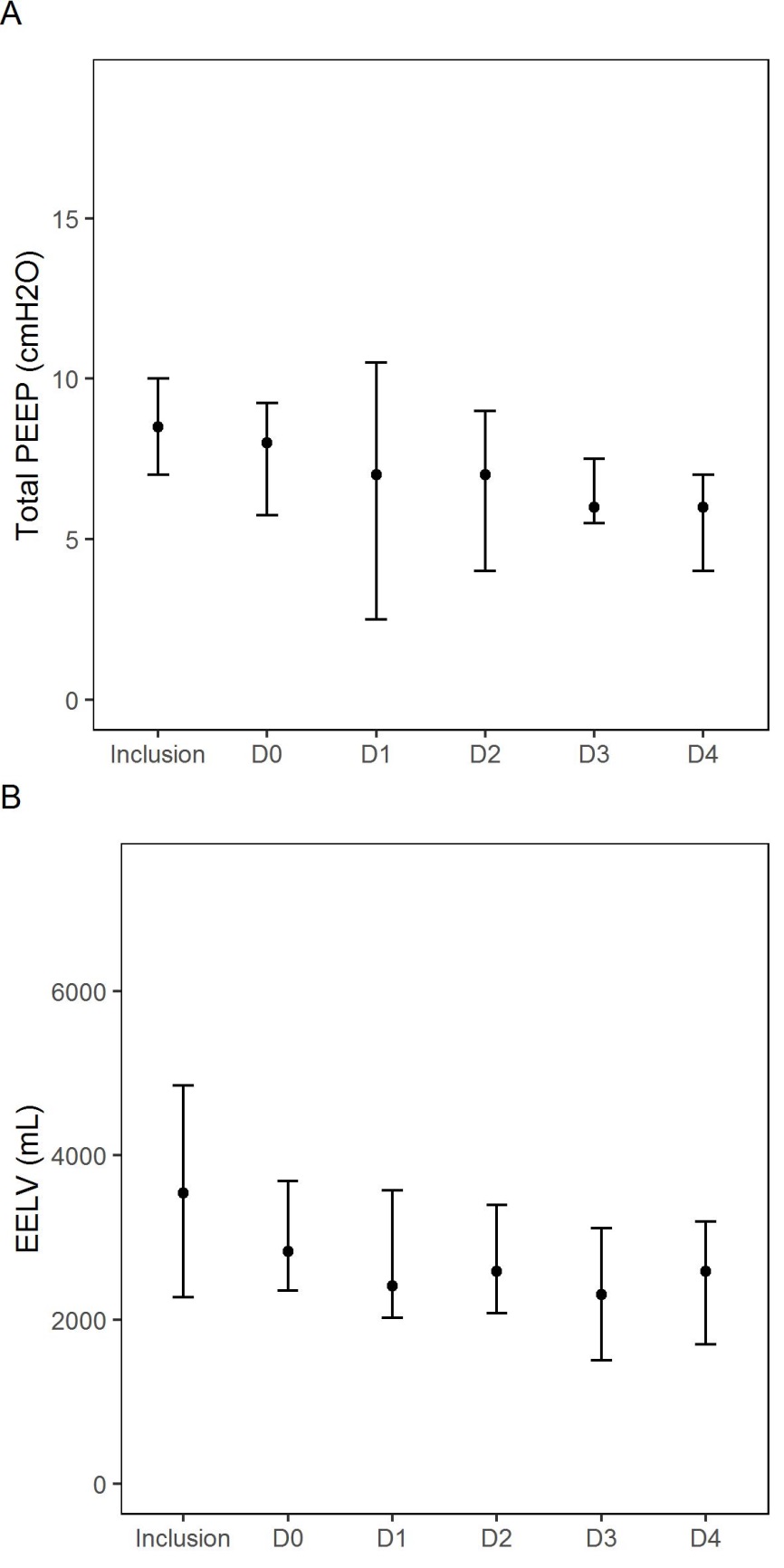


Panel A: total PEEP (end-expiratory occlusion) under ECCO_2_R until Day 4

Panel B: EELV (end-expiratory lung volume) under ECCO_2_R until Day 4

Figure 4esm: Daily course of ABG parameters under ECCO_2_R until Day 7.


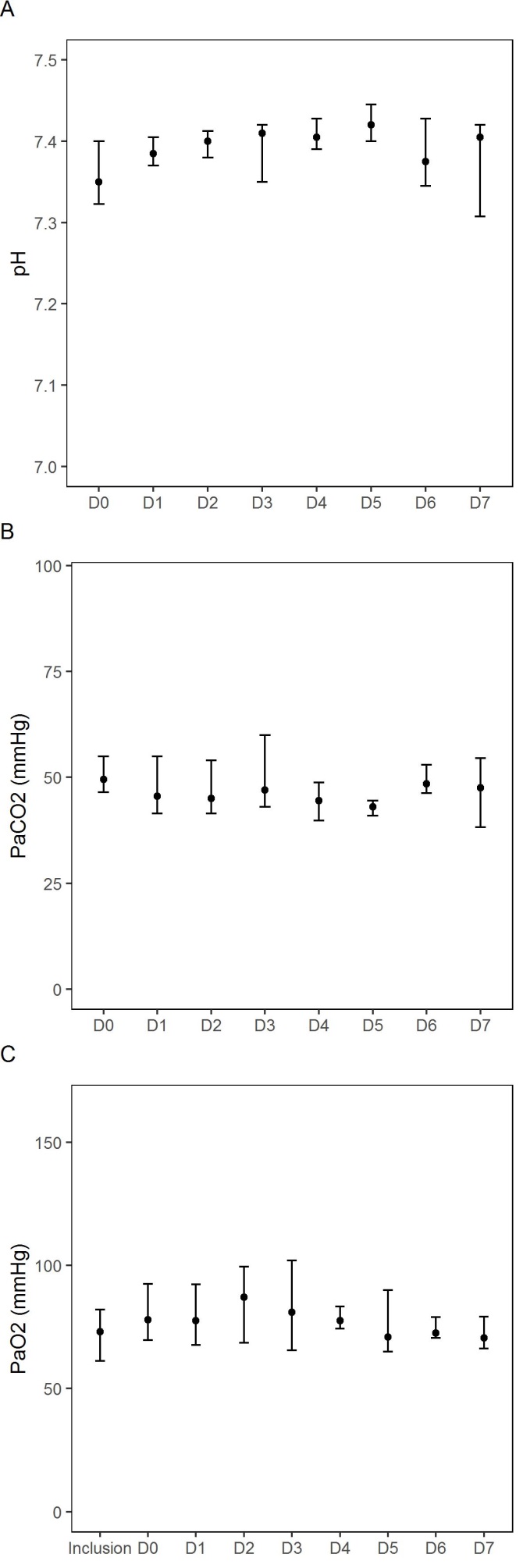


Panel A: pH: arterial pH value

Panel B: PaCO_2_: arterial CO_2_ partial pressure

Panel C: PaO_2_: arterial O_2_ partial pressure.

Figure 5esm: Course of hematological parameters under ECCO_2_R until Day 7.


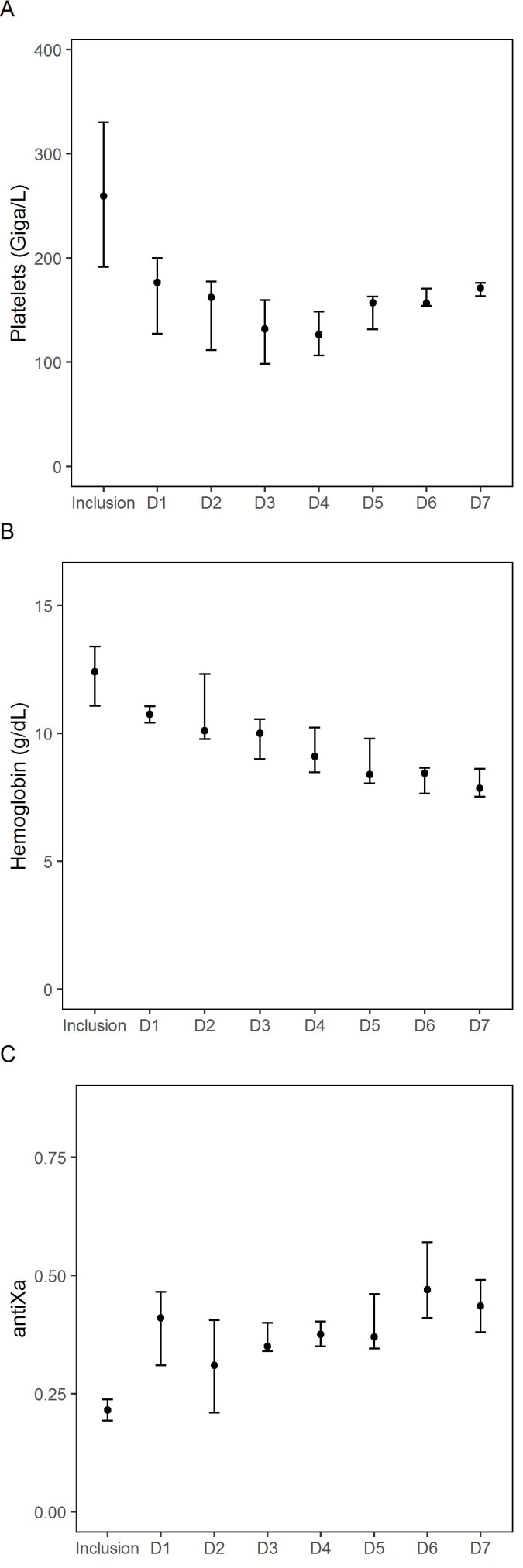


Panel A: Platelets

Panel B: Hemoglobin level

Panel C: antiXa activity calibrated
